# Therapeutic Gene Editing in Dyslipidemias

**DOI:** 10.31083/j.rcm2508286

**Published:** 2024-08-15

**Authors:** Seyed Saeed Tamehri Zadeh, Michael D. Shapiro

**Affiliations:** ^1^Prevention of Metabolic Disorders Research Center, Research Institute for Endocrine Sciences, Shahid Beheshti University of Medical Sciences, 19395-4763 Tehran, Iran; ^2^Center for Prevention of Cardiovascular Disease, Section on Cardiovascular Medicine, Wake Forest University School of Medicine, Winston Salem, NC 25157, USA

**Keywords:** dyslipidemia, gene editing, CRISPR/Cas9, *LDLR*, *PCSK9*, *ANGPTL3*, *APOC3*, Lp(a)

## Abstract

Dyslipidemia, characterized by abnormal lipid levels in the blood, significantly escalates the risk of atherosclerotic cardiovascular disease and requires effective treatment strategies. While existing therapies can be effective, long-term adherence is often challenging. There has been an interest in developing enduring and more efficient solutions. In this context, gene editing, particularly clustered regularly interspaced short palindromic repeats (CRISPR)/CRISPR-associated protein 9 (Cas9) technology, emerges as a groundbreaking approach, offering potential long-term control of dyslipidemia by directly modifying gene expression. This review delves into the mechanistic insights of various gene-editing tools. We comprehensively analyze various pre-clinical and clinical studies, evaluating the safety, efficacy, and therapeutic implications of gene editing in dyslipidemia management. Key genetic targets, such as low-density lipoprotein receptor (*LDLR*), proprotein convertase subtilisin/kexin type 9 (*PCSK9*), angiopoietin-like protein 3 (*ANGPTL3*), apolipoprotein C3 (*APOC3*), and lipoprotein (a) (Lp(a)), known for their pivotal roles in lipid metabolism, are scrutinized. The paper highlights the promising outcomes of gene editing in achieving sustained lipid homeostasis, discusses the challenges and ethical considerations in genome editing, and envisions the future of gene therapy in revolutionizing dyslipidemia treatment and cardiovascular risk reduction.

## 1. Introduction

Multiple lines of evidence, including animal studies, human genetics, epidemiology and randomized controlled studies, support the link between dyslipidemia and increased risk and severity of cardiovascular diseases (CVD) [1, 2]. Despite ongoing research to identify causes and treatments, dyslipidemia often remains underdiagnosed and inadequately treated [3]. It is often manageable through lifestyle modifications and medication, particularly statins. For patients at high risk, combining statins with other drugs like ezetimibe and proprotein convertase subtilisin/kexin type 9 (PCSK9) inhibitors may enhance treatment efficacy [4]. However, maintaining long-term medication adherence is challenging for many, leading to suboptimal outcomes [5]. Studies indicate that only about half of dyslipidemia patients continue with statin therapy, resulting in increased CVD incidents and healthcare costs [6]. Moreover, additionally, a significant number of patients with severe or refractory hypercholesterolemia, especially among certain women and ethnic groups, continue to exhibit high cholesterol levels despite maximum tolerated medical therapy, often due to drug side effects, non-adherence to treatment, or poor drug response [7, 8].

Advances in lipid therapeutics offer substantial hope for the future. The focus is increasingly shifting towards gene editing techniques [9]. Numerous *in vivo* studies have successfully modified the expression of liver-specific genes using these methods, and recent clinical trials have shown encouraging results in conditions like transthyretin amyloidosis. For instance, the use of NTLA-2001, a gene editing-based therapeutic, significantly slowed the progression of transthyretin amyloidosis in six patients [10]. While these findings are promising, the journey to integrate these therapies into clinical practice is long, considering the unknown effects and potential side effects. This review will delve into gene editing and its applications in dyslipidemia treatment, offering clinicians and researchers new insights to enhance patient care.

## 2. Gene Editing

The roles of genetic factors in a variety of diseases, such as cancers, infections, and inherited diseases, are well-established. In response, scientists have been diligently working to develop new genetic tools. These tools aim to bolster immune reactions against harmful cancers, deactivate the genetic material of disease-causing pathogens, and rectify genetic mutations. While introducing new genes has shown promise, gene editing is often crucial for achieving therapeutic objectives.

Genome editing has become a focal point of interest due to its ability to create a range of genetic modifications across different settings. Recent scientific and technological advancements have led to the development of various gene editing methods. These include transcription activator-like effector nucleases (TALEN), homologous recombination, zinc-finger nucleases (ZFNs), and, most notably, the recent innovation of clustered regularly interspaced short palindromic repeats-clustered regularly interspaced short palindromic repeats (CRISPR)-associated protein 9 (CRISPR-Cas9).

CRISPR-Cas9 has emerged as the most popular method due to its effectiveness and versatility. However, despite its numerous advantages, CRISPR-Cas9 is not without limitations.

## 3. Genome Editing Technologies

Base editing, epigenome editing, and prime editing are molecular techniques that utilize the high precision of the CRISPR/Cas9 system to target specific genomic sites accurately. The capacity to target specific regions of DNA stays intact even after alterations are made to the Cas9 cleavage domains. These modifications give rise to two variants of Cas9: nickase Cas9 (nCas9), which cleaves just one strand of DNA, and dead Cas9 (dCas9), which does not cleave DNA. The introduction of extra domains to nCas9 or dCas9 can enhance the capabilities of the CRISPR/Cas9 system by including a range of activities.

### 3.1 Nuclease Editing

The CRISPR/Cas systems, originating in bacteria, consist of two main varieties: CRISPR/Cas9 and CRISPR/Cas12. These variants function as platforms for editing genomes. The fundamental constituents of these systems consist of a Cas protein and a guide RNA [11]. The guide RNA exhibits inherent search and binding capabilities built into its RNA sequence. On the other hand, the Cas protein is responsible for coordinating the initiation of a DNA double-strand break, utilizing one or two cleavage domains to cleave both strands of DNA. The primary utilization of the CRISPR/Cas system for genome manipulation in mammalian cells entailed the utilization of Streptococcus pyogenes Cas9, generally denoted as SpCas9 [12, 13, 14]. The guide RNA, approximately 100 nucleotides long, contains information that determines the specificity of DNA targeting. This information is encoded within the first 20 nucleotides of the guide RNA, referred to as the spacer region. The SpCas9 protein establishes a molecular complex with RNA, specifically binding to the remaining 80 nucleotides. The protein-RNA complex engages in the process of scanning double-stranded DNA molecules that it comes into contact with. During DNA scanning, the SpCas9 enzyme exhibits transient pauses at particular areas with NGG patterns, where the letter N denotes any nucleotide. Currently, the guide RNA’s spacer region exhibits alignment with the DNA strand that does not possess the NGG pattern, commonly called the target strand. The occurrence of a strong affinity between the target strand and spacer sequences leads to a notable formation of Watson-Crick base pairs between DNA and RNA, thereby initiating the activation of SpCas9. As a result, a double-strand break arises close to the third base pair preceding the NGG motif. The DNA sequence located on the non-target strand corresponding to the RNA spacer sequence is called the protospacer. This protospacer includes a 20-nucleotide region positioned directly upstream of the NGG motif, known as the protospacer-adjacent motif (PAM).

### 3.2 Base Editing

**Fig. 1.**
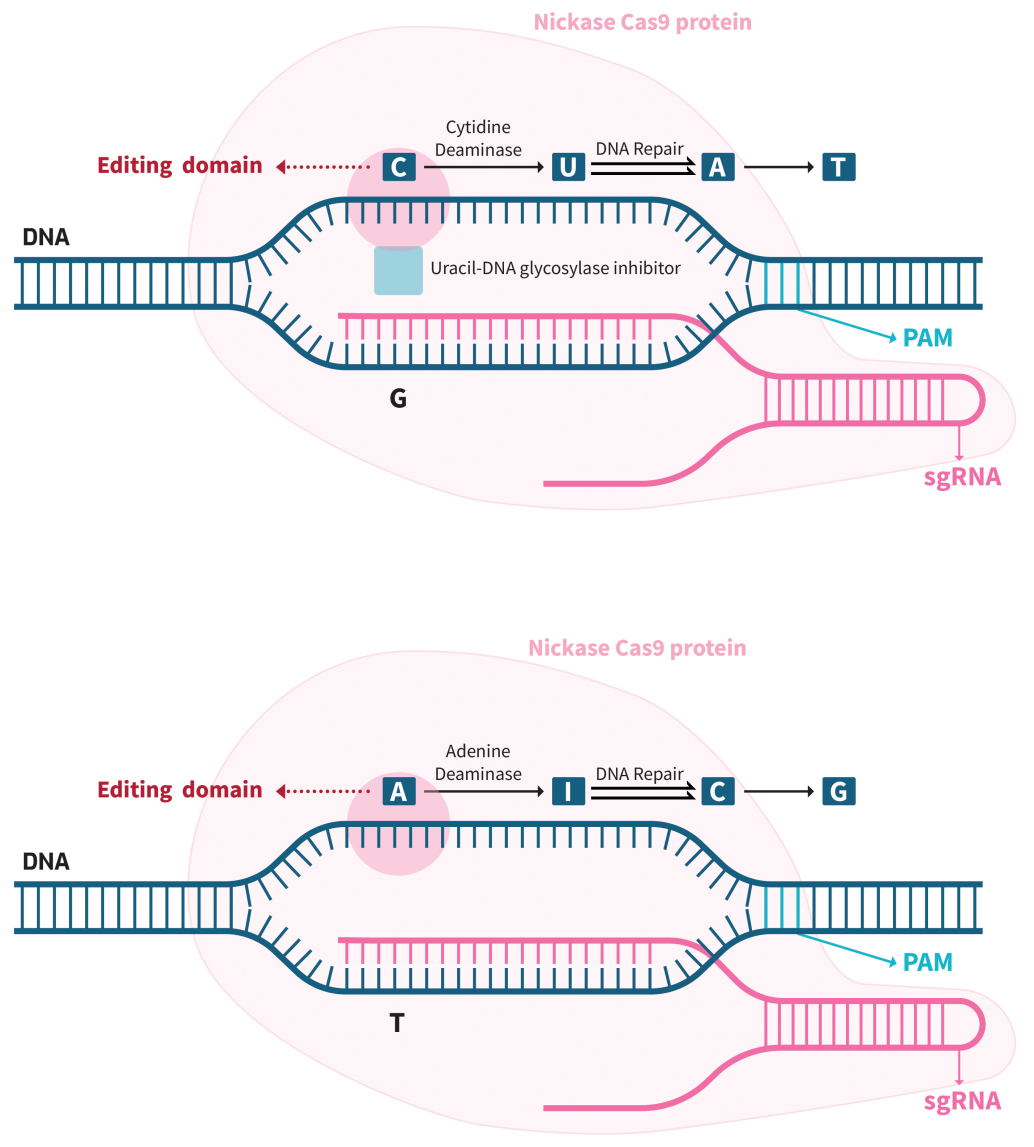
**CRISPR DNA base-editing tools**. A: DNA base-editors have two main components, which are a Cas enzyme for programmable DNA binding and a base modification enzyme for targeted nucleotide change. Two types of DNA base-editors have been developed: cytosine base-editors and adenine base-editors. Cytosine base editing: cytosine deaminase produces uracil, that base pairs as thymidine; uracil-DNA glycosylase inhibitor enhances the efficacy of cytosine base-editing by binding and inhibiting uracil N-glycosylate: adenine base editing: adenine deaminase generates inosine, that base pairs as thymidine (modified from Kantor *et al*. [18]). PAM, protospacer adjacent motif; sgRNA, small guide RNA; A, adenine; C, cytosine; T, thymine; G, guanine; U, uracil; I, inosine; Cas9, clustered regularly interspaced short palindromic repeats (CRISPR)-associated protein 9.

There are two main classifications of base editors: cytosine base editors, which assist the conversion of a cytosine (C) base to another base, often thymine (T) or occasionally guanine (G) [15, 16], on a DNA strand, and adenine base editors, which enable the replacement of an adenine (A) base with a guanine (G) [17]. The capability to catalyze cytidine residue deamination inside a specified editing window on the non-target DNA strand can be achieved by combining nCas9 with various cytidine deaminase domains sourced from natural origins, such as apolipoprotein B mRNA editing enzyme catalytic subunit 1 (APOBEC1) or activation-induced cytidine deaminase (AID) proteins. The DNA strand separation facilitated by Cas9, followed by the binding of the target strand to the guide RNA, results in an R-loop structure. The arrangement of this structure leads to the creation of a DNA bubble that consists of a single strand inside a certain region of the non-hybridized and non-targeted strand. This region is easily accessible to the deaminase domain, which determines the editing window. The dimensions of the editing window are contingent upon the particular Cas9 ortholog utilized, as deamination results in the conversion of C to U (uracil). The enzyme uracil-DNA glycosylase is commonly involved in the reversal of this conversion. However, the introduction of an inhibitor domain to nCas9 hinders the occurrence of this repair process. The nCas9 enzyme exhibits high specificity in its ability to cleave the target strand. Simultaneously, the repair mechanism entails the removal of nucleotides surrounding the nick site and their subsequent replacement by complementary base pairing with the non-target strand. The presence of a U nucleotide in the non-target strand induces the placement of an A nucleotide in the corresponding position of the target strand, as a result of the complementary base pairing between A and U. The process of cellular repair involves the replacement of the non-standard U nucleotide, typically present in RNA, with the ordinary T nucleotide. As a result, a cytosine-guanine (C-G) base pair is converted into a thymine-adenine (T-A) base pair (Fig. 1, Ref. [18]) [19].

### 3.3 Prime Editing

Prime editing involves the combination of Cas9 nickase with a specifically engineered reverse transcriptase enzyme. This enzyme utilizes an RNA template to aid in incorporating new DNA sequences. This methodology functions autonomously from DNA double-strand breaks or the presence of a donor DNA template, hence enabling precise incorporation of targeted point mutations or indels across a wider range of editing possibilities. The application of prime editing has been utilized in laboratory settings to correct a variety of genetic disease-causing factors in human cell lines. This has led to the development of specific therapeutic methods tailored to each target gene [20]. Although there is currently minimal *in vivo* testing of this approach, it has the potential to effectively target a wide range of mutations, such as deletions, duplications, and inversions. As a result, it presents intriguing opportunities for correcting most disease-causing variations. In addition, a primary editing guide RNA (pegRNA) facilitates the accurate identification and targeting of vulnerable gene areas, hence enabling the correction of closely located genetic variations within such regions. As mentioned above, the capability surpasses the limitations of single-base editing. It demonstrates superior efficacy compared to homology-directed repair (HDR), significantly expanding the range of sequence targets and therapeutic approaches [21].

### 3.4 Epigenome Editing

Epigenome editing distinguishes itself from other editing modalities by confining alterations solely to DNA sequences. In contrast, it influences gene expression by modulating protein-DNA interactions. When a compound consisting of dCas9 and guide RNA (gRNA) is targeted towards a specific region located within a gene promoter or transcriptional enhancer, it indirectly influences the DNA molecule. However, it can impede the expression of genes through a process known as CRISPR interference, which hinders the interactions between certain components that would normally bind to the specific sequence being targeted [22]. The knockdown effectiveness can be augmented by fusing dCas9 with a gene expression-inhibiting domain, such as the Krüppel-associated box (KRAB) domain. The fusion event leads to a restructuring of the chromatin structure in the vicinity, resulting in a modification of the accessibility of the DNA sequence for the transcriptional machinery [23]. In contrast, the augmentation of gene expression through CRISPR activation can be achieved by combining dCas9 with a transcriptional activator domain, such as VP16, or by connecting activators to the gRNA via the manipulation of RNA aptamers located at its 3’ terminus [24]. It is worth noting that the observed changes in gene expression are temporary, occurring only while the dCas9 fusion protein is present at the designated location. To obtain long-term stability in the process of epigenome editing, it is possible to lengthen the duration of activity by combining dCas9 with a methyltransferase or demethylase domain [25]. Enhanced methylation in the vicinity of the promoter region of a certain gene, particularly at cytosine bases within CpG dinucleotide sequences, typically results in the inhibition of gene expression. In contrast, a reduction in methylation generally increases gene expression. Persistent methylation alterations have been seen to endure during numerous cellular divisions. However, it is possible to undo these modifications by utilizing a fusion protein of dCas9 that has an opposing influence on the same genomic location [26].

## 4. Gene Editing for the Management of Dyslipidemia

Gene editing techniques have been employed to either disrupt or repair targeted genes, garnering significant attention for their potential in managing dyslipidemia. So far, this approach has yielded encouraging results. While gene repair is primarily applicable to the treatment of monogenic diseases, gene disruption offers broader possibilities in this context. A variety of genes that play pivotal roles in lipid metabolism, such as *low-density lipoprotein receptor* (*LDLR*), *PCSK9*, *angiopoietin-like protein 3* (*ANGPTL3*), *apolipoprotein C3* (*APOC3*), and lipoprotein(a) (*LPA*), have been strategically targeted for the treatment of dyslipidemia, showcasing the versatility and promise of gene editing in this field.

### 4.1 LDLR 

Familial hypercholesterolemia (FH) is the most common monogenic disorder in humans. It follows an autosomal dominant inheritance pattern andis characterized by tendinous xanthomas, arcus juvenilis, and premature coronary heart disease (CHD) [27]. FH is due to mutations in three canonical genes, which encode *LDLR*, apolipoprotein B (*apoB*), or *PCSK9*, accounting for approximately 90%, 5%, and 1% of all patients, respectively. The *LDLR*, which is located on the cell surface, is expressed in hepatocytes and contributed to regulating LDL levels [28].

Heterozygous mutations can cause CVD in middle age, and currently, available treatments, such as statins, work well in these patients, however low density lipoprotein cholesterol (LDL-C) is often suboptimally lowered with statin monotherapy. However, treatments are minimally effective in those with homozygous mutations with FH (HoFH) [29]. Statins decrease the blood cholesterol and LDL-C via inhibiting hydroxy-methyl-glutaryl coenzyme-A (HMG-CoA) reductase and increasing the expression of *LDLR*, leading to enhance in LDL-C removal from the circulation [30]; thus, the therapeutic effects of statins hugely depend on the* LDLR* function and those with non-functional* LDLR* do not respond on statin therapy [31]. Homozygous individuals are prone to develop CVDs at early stages of life, contributing to early premature death [32, 33].* LDLR* is virtually absent in HoFH patients. Therefore, available treatments cannot reduce LDL levels efficiently [34]. To prolong the survival of these patients, we should use more aggressive approaches, including combination lipid lowering therapy. Apheresis is also an option but is demanding for patients, expensive, and availability is limited [35].

Carlson *et al*. [36] used TALENs to inactivate both alleles of the *LDLR* gene in the pig fibroblast genome, which was obtained in 10 out of 11 piglets following somatic cell nuclear transfer. Hence, they concluded that in the biomedical field, TALENs, which can be accessed very readily in all labs, have the potential for generating lipid metabolic disease [36]. In another experimental research, to mimic lipid metabolic disease, Huang *et al*. [37] targeted both *LDLR* and *ApoE* genes of porcine embryonic fibroblasts at the same time through applying the CRISPR/Cas9 method. All six founder pigs displayed biallelic inactivation of *LDLR* without finding off-target incidents and serum values of cholesterol, LDL, and ApoB were enhanced remarkably, indicating that CRISPR/Cas9 approach can be utilized to simulate lipid metabolic disease and atherosclerotic models.

In recent years, gene therapy has been considered a potential solution for the management of HoFH patients. Omer *et al*. [38] cultured skin fibroblasts from a HoFH patient with 3-base pair deletion in exon four of *LDLR* and thereafter, to correct mutations permanently, the investigators used Cas9 nickase (Cas9n) with paired single-guide RNAs (sgRNA). The insertion corrected mutations successfully and receptor function was restored. It was found that both alleles of 83% (10 out of 12) of enriched clones were corrected. Moreover, these reprogrammed cells were capable of differentiating into hepatocyte-like cells, which were shown to have a comparable LDL endocytosis compared to hepatocytes cells with normal *LDLR* function. Therefore, by applying this method, we can have access to countless source of autologous hepatocytes that either can be delivered to the liver directly or engineered into a functional liver-like tissue, helping us to restore the normal function of *LDLR* and reduce LDL levels [38].

In another research by Jarrett *et al*. [39], an *in vivo* model of metabolic diseases was generated using the adeno-associated virus (AAV)-CRISPR approach. In order to generate the disease, the authors introduced guide RNAs, which were able to target exon 14 of *LDLR* or exon 5 of *ApoB*. *LDLR* disruption contributed to severe hypercholesterolemia and atherosclerosis. On the contrary, mice with disruption of both *LDLR* and *ApoB* showed a rapid decrease in plasma cholesterol, which lasted for 20 weeks, indicating that ApoB disruption can decrease cholesterol levels significantly. In addition, while atherosclerotic plaques were easily detectable in mice with* LDLR* disruption, concomitant disruption of *ApoB* and *LDLR* exerted anti-atherosclerotic effects. Although ApoB disruption showed promising outcomes in terms of reducing plasma cholesterol, hepatic fat accumulation was observed, which can be detrimental and limit the applicability of this method in clinical practices. Another limitation of this approach was the high rates of off-target events [39].

Very recently, Zhao *et al*. [40] designed an *in vivo* experiment to evaluate CRISPR/Cas9 delivered by AAV as a method of targeted genome editing to restore the function of LDLR and improve atherosclerosis in cases with HoFH. They produced a mouse model of atherosclerosis, *Ldlr^E⁢208⁢X^*, by inserting a nonsense mutation. They sought to correct the mutation in the *LDLR* gene using CRISPR/Cas9 and subsequently, injected these cells into mice. The researchers demonstrated that the function of LDLR protein was restored partially (6.7%) (the restoration was defined by homology-directed repair-mediated correction of the T-G mutation), while the LDLR protein level was restored to roughly 18% in six wild type mice. Treated mice had lower serum levels of triglyceride, cholesterol (65%), and LDL, as well as lower macrophage infiltration and smaller atherosclerotic plaques compared to the control group. Thus, AAV-CRISPR/Cas9–mediated *LDLR* gene correction can be taken into account for HoHF treatment [40].

Satisfactory findings regarding targeting *LDLR* have been achieved (Table 1, Ref. [36, 37, 38, 39, 40]), which can make it a potential target for the treatment of HoHF patients; however, there is a main challenge that should be mentioned. Nearly 2000 pathogenic variants of *LDLR* have been identified so far [41], and it seems profoundly difficult to evaluate the value and safety of current personalized approaches in clinical settings using available technologies. Therefore, it is better to take into account other available options to change the values of LDL and ameliorate CVDs.

**Table 1.** **Comparative overview of gene editing interventions on LDLR**.

Author	Study design	Method	Findings	Treatment-related adverse effects
Carlson *et al*. [36]	Pig	TALEN	Biallelic inactivation of* LDLR* in 10 out of 11 animals	-
Huang *et al*. [37]	Pig	CRISPR/Cas9	Biallelic inactivation of *ApoE* and *LDLR* in all animals	No off-target mutations were found.
			Elevated plasma levels of LDL-C, ApoB, and TC	
Omer *et al*. [38]	Human iPSC	CRISPR/Cas9	Both alleles of 83% of enriched clones were corrected	No off-target mutations were detected.
Jarrett *et al*. [39]	Cas9 transgenic mice	AAV-CRISPR	*LDLR* disruption led to hypercholesteremia and atherosclerosis	Off-target sites were found for the *LDLR* gene, but not *ApoB*.
			*ApoB* disruption led to lower levels of cholesterol	Mice received LDLR + ApoB gRNAs showed evidence of microvesicular steatosis.
Zhao *et al*. [40]	*Ldlr^E⁢208⁢X^* mouse	AAV-CRISPR/Cas9	*LDLR* expression restored partly	Most of off-target mutations were found in introns of different genes.
			Lowered plasma levels of cholesterol, TG, and LDL-C	

LDLR, low density lipoprotein receptor; CRISPR, clustered regularly interspaced short palindromic repeats; Cas9, CRISPR-associated protein 9; TALEN, transcription activator-like effector nuclease; ApoB, apolipoprotein B;* ApoE*, apolipoprotein E; LDL-C, low density lipoprotein cholesterol; TG, triglyceride; iPSC, induced-pluripotent stem cell; AAV, adeno-associated virus; gRNAs, guide RNAs; TC, total cholesterol.

### 4.2 PCSK9

It has been demonstrated that PCSK9 has crucial roles in degrading LDLR in hepatocytes. It can bind to LDLR and this complex prevents LDLR from entering recycle vesicles, preventing LDL from returning to the cell surface. Formation of this complex in the Golgi apparatus can enhance the transfer of the complex from the Golgi apparatus toward the lysosome, leading to a remarkable increase in LDLR degradation [42].

There is accumulated evidence in support of the fact that* PCSK9* can be considered a promising potential therapeutic target for dyslipidemia and CVDs [43]. It has been repeatedly demonstrated that the loss-of-function mutations in *PCSK9* are associated with lower LDL and coronary events and no adverse effects [44, 45]. Consequently, several medications with the potential of inhibiting PCSK9 have yielded hopeful results. Evolocumab was introduced as a monoclonal antibody that prohibits PCSK9 and reduces LDL up to 60%, resulting in some benefits for those with CVDs [46]. Another human monoclonal antibody is alirocumab which can reduce the risk of CVD events in people with a history of acute coronary syndrome [47]. Several studies have investigated the applicability of genome editing for managing dyslipidemia via targeting *PCSK9* and here we discuss some of these investigations.

Up to now, several studies have assessed the applicability of gene editing to target the *PCSK9* gene (Table 2, Ref. [48, 49, 50, 51, 52, 53, 54, 55, 56, 57, 58, 59, 60]). The first *in vivo* study that sought to disrupt the expression of *PCSK9* in mice was conducted by Ding *et al*. [48]. The authors applied adenovirus to deliver CRISPR/SpCas9 and a guide RNA to target *PCSK9* accurately. Four days after the injection, they found over 50% mutagenesis in *PCSK9* in all mice, which led to a substantial reduction in plasma *PCSK9*, augmented LDLR levels, and a 35%–40% decrease in plasma cholesterol values. Interestingly, off-target incidents were not observed in ten sites and another study found no off-target incidents in up to 180 sites using the CRISPR/SpCas9 approach [48]. While adenovirus can deliver SpCas9 efficiently, it is noteworthy to mention that it can induce an immune response [61]. Moreover, delivering SpCas9 due to its size, limits its usage in experimental research. Consequently, scientists introduced AAV as a more proper tool because it provokes immune response less [62] and has a wider range of serotype specificity [63]. In this regard, Ran *et al*. [49] designed six Cas9 from *Staphylococcus aureus* (SaCas9), which is 1 kb shorter than SpCas9, enabling the packing of sgRNA into an AAV with a limited cargo size. The investigators made a package consisting of sgRNA, SaCas9, and AAV *in vitro*, and injected that package to target the *PCSK9* of mice liver. After 7 days, they found over 40% of disruption in the *PCSK9* gene, contributing to approximately 95% and 40% reduction in the serum values of PCKS9 and cholesterol, respectively. In addition, they evaluated the sensitivity and specificity of SaCas9, which were promising [49]. In order to address the main drawback of using viruses as a delivery tool, Yin *et al*. [50] incorporated the combination of SpCas9 and modified sgRNA into a lipid nanoparticle. Modification of sgRNA did not prohibit the interaction between sgRNA and SpCas9, and increased genome editing activity. Five days following the intravenous injection, it was observed that up to 80% of the *PCSK9* gene was disrupted, serum PCSK9 protein values were undetectable, and cholesterol levels were reduced by 35%–40% in mice [50].

**Table 2.** **Comparative overview of gene editing interventions on PCSK9 and cholesterol levels**.

Author	Study design	Method	Findings	Treatment-related adverse effects
Ding *et al*. [48]	C57BL/6 mice	CRISPR-SpCas9	50% mutagenesis in *PCSK9*	No off-target mutations were detected.
			Decreased plasma levels of PCSK9 and cholesterol	
			Increased hepatic levels of LDLR	
Ran *et al*. [49]	C57BL/6 mice	CRISPR/SaCas9	Decreased serum levels of PCSK9 and total cholesterol	No off-target mutations were detected.
Yin *et al*. [50]	C57BL/6 mice	Nanoparticle-CRISPR-SpCas9	80% editing in *PCSK9* gene	No off-target mutations were detected.
			Very low serum levels of PCSK9	
			35–40% reduction in serum levels of cholesterol	
Wang *et al*. [51]	Chimeric liver-humanized mice	Chimeric liver-humanized mice model	52% reduction in PCSK9 protein levels	No off-target mutations were detected.
Chadwick *et al*. [52]	C57BL/6 mice	Adenoviral base editing (BE3)	Decreased plasma levels of PCKS9 protein (50%) and cholesterol (30%)	No off-target mutations were detected.
Levy *et al*. [53]	C57BL/6 mice	AAV-cytosine and adenine base editing	38% genome editing in the liver	A single off-target site.
Musunuru *et al*. [54]	Cynomolgus monkeys	ABE8.8-m nanoparticle- base editing	Decreased plasma levels of PCSK9 protein (90%) and LDL-C (60%)	No off-target editing with a dose of 0.5 mg/kg LNP and a low level of target editing (mean <1%) with a dose of 1.5 mg/kg LNP.
Wang *et al*. [55]	Rhesus macaque	AAV-meganuclease editing	Decreased plasma levels of PCSK9 protein (84%) and LDL-C (60%)	First generation: 487–629 off-target cleavage sites were identified.
				Second generation: 192–257 off-target cleavage sites were identified.
Breton *et al*. [56]	Rhesus macaque	AAV-meganuclease editing	Reduction in plasma levels of PCSK9 (40–76% of baseline) and LDL-C (64–89% of baseline)	Off-target editing, which was lower in the second generation of AAV-meganuclease editing compared to the first generation.
Wang *et al*. [57]	Rhesus macaque	AAV-meganuclease editing	Decreased plasma levels of PCSK9 and LDL-C, which consistently lasted for three years	Mild-to-moderate liver capsular and subcapsular fibrosis and minimal-to-mild mononuclear cell infiltrates in the liver tissue.
				A low frequency of off-target editing.
Rothgangl *et al*. [58]	C57BL/6 and Alb-Cre × Trp53^flox/flox^, cynomolgus macaques	ABE base editing	Mice: Plasma levels of PCSK and LDL-C decreased 95% and 58%, respectively	No off-target genome editing in DNA.
			Macaques: Plasma levels of PCSK and LDL-C decreased 32% and 14%, respectively	Very mild lobular mixed cell infiltration in the liver.
Lee *et al*. [59]	Cynomolgus monkeys	ABE8.8-m nanoparticle- base editing	Mean *PCSK9* editing of 46% and 70% following treatment with *VERVE-101* at 0.75 and 1.5 mg/kg, respectively	A transient rise in alanine aminotransferase and aspartate aminotransferase, which resolved after two weeks.
			67% and 83% reductions in the blood levels of PCSK9, and 49% and 69% reductions in blood levels of LDL-C, which lasted for 476 days, following treatment with 0.75 and 1.5 mg/kg *VERVE-101*, respectively	No change in total bilirubin.
Bellinger [60]	10 HeFH patients with a history of coronary revascularization and a mean LDL-C of 193 mg/dL	ABE8.8-m nanoparticle- base editing	PCSK9 levels were decreased by 59% and 84% in two patients received 0.45 mg/kg dose and 45% in the patient received 0.6 mg/kg	One patient experienced a fatal cardiac arrest 5 weeks after infusion.
			LDL-C level was reduced by 39% and 48% in participants received 0.45 mg/kg dose and 55% in the participant received 0.6 mg/kg dose	One patient experienced myocardial infarction one day after drug infusion and non-sustained ventricular tachycardia 4 weeks after infusion.
			A 55% reduction in LDL-C of the patient received 0.6 mg/kg persisted for six months	

*LDLR*, low-density lipoprotein receptor; PCSK9, proprotein convertase subtilisin/kexin type 9; CRISPR, clustered regularly interspaced short palindromic repeats; SpCas9, Streptococcus pyogenes Cas9; Cas9, CRISPR-associated protein 9; LDL-C, low density lipoprotein cholesterol; AAV, adeno-associated virus; ABE, adenine base editors; HeFH, heterozygous familial hypercholesterolemia; LNP, lipid nanoparticle.

Base editing enables scientists to accurately introduce single-nucleotide variants into disease-causing genes and modify their function [54]. For the first time, Chadwick *et al*. [52] examined the therapeutic potential of base editor 3 (BE3), as the most commonly used base editor, to generate nonsense variants in *PCSK9* liver tissues of mice. This approach led to decreased plasma PCSK9 protein and cholesterol by approximately 50% and 30%, respectively, and no off-target incidents were found. Although its efficacy was lower than genome editing, it was associated with a lower risk of finding on-target indels and off-target incidents because this process does not need to break double-strand DNA. One of the main drawbacks of this approach is using adenovirus for delivering base editor because BE3 is large [52]. To tackle the size limitation, Levy *et al*. [53] used a split base-editor dual-AAV approach as a more suitable vector and revealed the efficacy of base editing in the mice liver was 38%.

In another experimental study, adenine base editor was delivered via lipid nanoparticles to disrupt the expression of *PCSK9*. First, on hepatocytes, the levels of splice site editing were measured up to 60% and *PCSK9* declined about 55%. Thereafter, 70% based editing at the splice site was detected in the liver of mice. In the final step, the authors assessed the efficacy of this approach on monkeys. It was shown that PCSK9 protein and cholesterol levels decreased by about 81% and 65%, respectively, suggesting that the approach could edit both *PCSK9* alleles of virtually all hepatocytes. Using lipid nanoparticles provoked a mild to moderate rise in liver function tests (aspartate aminotransferase (AST) and alanine aminotransferase (ALT)) which lasted for only 1–2 weeks, whereas using AAV meganuclease can cause a moderate rise in these enzymes, which lasts for months. Off-target editing was found at one site of monkeys, which shows no similar homology to the human genome, which is consistent with the fact that no off-target editing was found in human hepatocytes [54]. The efficacy of the approach in terms of reducing LDL was similar to or better than lipid-lowering agents, and a 65% reduction in LDL can reduce the risk of CVD substantially [64]. Similar to the last study, Rothgangl *et al*. [58] utilized an adenine base editor that was delivered by a lipid nanoparticle, comprising of a gRNA and the base editor messenger RNA (mRNA). The authors used adenine base editing to target splice donor site of *PCSK9* intron 1. First, they tested the efficacy of RNA on mice, and found a significant decrease in the levels of PCSK9 and LDL-C. Thereafter, 0.75 mg/kg or 1.5 mg/kg RNA was injected in four groups of macaques. Each dose was given as a single dose or as two doses after two weeks from the first dose. After a month, base editing was occurred in 2.03 ± 0.85% and 27.6 ± 5.87% of groups received a single dose of 0.75 and 1.5 mg/kg, respectively. The corresponding values for groups receive two doses of 0.75 and 1.5 mg/kg were 3.31 ± 1.73% and 24.14 ± 1.52%, respectively. The second injection did not enhance the rate of base editing, because after the first injection, IgG antibody against base editor was formed. In animals received 1.5 mg/kg RNA, PCSK9 levels were reduced about 26% and 39% in the single- and repeated-dose group, respectively, which was associated with a 9% and 19% reductions in the levels of LDL-C in the single- and repeated-dose group, respectively. No off-target mutations were detected [58].

Although these research have demonstrated the effect and safety of *PCSK9* editing in mice, the translation of these findings into human patients is limited due to several reasons. First of all, off-target incidents observed in mice models probably not hold up in the human genome since there are notable differences between genomes of a mouse and a human. Secondly, if the same site in the mouse and human genome being targeted using the same sgRNA, the outcomes of *PCSK9* editing will be hugely different as human and mouse hepatocytes are physiologically different [26]. In order to overcome this obstacle, Wang *et al*. [51] designed chimeric liver-humanized mice model in which mice hepatocytes were replaced by human hepatocytes in order to target the human *PCSK9* gene. Blood values of PCSK9 protein were reduced on average by 52% and no off-target incidents were reported [51].

To translate the efficacy of genome editing into clinical settings, it is mandatory to assess their performance on animal models other than mice. Wang *et al*. [55] revealed that meganuclease targeting of *PCSK9* in the liver of primates brings about 84% and 60% reduction in serum levels of PCSK9 and LDL, respectively. Of note, this approach had some consequences that should be mentioned. First, off-target editing was remarkably high, which can lead to the translocation of chromosomes. Second, a mild rise in AST and ALT was found, which lasted for approximately 40 days [55]. In order to limit off-target mutations, the same authors performed two different strategies: (1) a shorter version of thyroid hormone-binding globulin (TBG) to decrease nuclease expression, (2) inserting the *M2PCSK9* target sequence into the AAV genome that expresses the nuclease and/or fusing the nuclease to a specific peptide to facilitates its degradation. The investigators observed the same efficacy with the less off-target mutations [56]. In the later study, they found that the impact of this method on PCSK9 and LDL-C can last for three years consistently [57]. These findings highlight the importance of this approach for the treatment of those with genetic disorders because these patients need treatments with life-long effects. Little off-target editing was found and they were stable during the follow-up.

Promising findings concerning the efficacy, safety, and durability of a new drug, called *VERVE-101*, for reducing LDL-C levels has brought hope for future management of dyslipidemia using genome editing. *VERVE-101*, an investigational CRISPR base-editing medicine, was designated to reduce LDL-C in patients with FH. This medicine consists of an engineered lipid nanoparticle that encapsulates an adenine base editor messenger RNA that Verve has licensed from Beam Therapeutics, and a guide RNA targeting the *PCSK9* gene [65]. The drug comprises of a guide RNA that targets a splice donor at the boundary of intron 1 and exon 1 of *PCSK9* and introduce a stop codon adjacent to the beginning of intron 1, which would terminate the generation of a PCSK9 protein and the mRNA with the ability to encode the adenine base editor 8.8-m [66]. Musunuru *et al*. [54] found a near-complete knockdown of *PCSK9* in the livers of cynomolgus monkeys following a single infusion of *VERVE-101*. They observed reductions in blood levels of PCSK9 and LDL-C by about 90% and 60%, respectively, which were remained for at least 8 months following the administration. Its efficacy in terms of reducing LDL-C levels equals or surpasses the efficacy of existing treatments and moreover, in contrast to other treatments, *VERVE-101* can offer once-and-done treatment of enhanced levels of LDL-C. A very recent study conducted by Lee *et al*. [59] sought to evaluate the efficacy, durability, and safety of *VERVE-101* among cynomolgus monkeys. Authors decided to choose 1.5 mg/kg *VERVE-101* for the experiment since they are closely related to humans in terms of genetic and physiology. Additionally, authors did not perform any modification on the drug since the target DNA location for the drug was similar between humans and cynomolgus monkeys. 36 cynomolgus monkeys were employed of which 10 received a vehicle control, four received 0.75 mg/kg *VERVE-101*, and 22 received 1.5 mg/kg *VERVE-101*. The mean editing of *PCSK9* was measured two weeks after the dosing using targeted amplicon sequencing method and was 0.1%, 46%, and 70% in the control group, the group received 0.75 mg/kg, and the group received 1.5 mg/kg, respectively. Animal treated with 0.75 mg/kg experienced 67% and 49% reductions in the levels of PCSK9 and LDL-C, respectively. The corresponding values for animals treated with 1.5 mg/kg were 83% and 69%, respectively. Dose-dependent and durable (up to 476 days) decrease in the levels of PCSK9 and LDL-C was detected; percentage changes for PCSK9 from baseline values were 8%, 49%, and 69% for the control group, animals received 0.75 mg/kg, and animals received 1.5 mg/kg, respectively; the corresponding values for LDL-C were 10%, 50%, and 68%, respectively. The investigators found a strong correlation between the levels of PCSK9 and LDL-C 12 months after dosing. Regarding safety, a transient rise in aspartate AST and ALT following infusion, which were resolved after two weeks. 12 months after dosing, histopathological evaluations of liver and other organs of subjects did not show any microscopic and macroscopic changes related to the drug. No significant change in the level of *PCSK9* editing before and 3 months after dosing was found. The *PCSK9* edit was not transmit to any offsprings [59].

These preclinical efficacy and safety data contributed to the initiation of an ongoing human clinical trial, namely heart-1. Heart-1, an open-label, single ascending dose trial, was designed to examine the efficacy and safety of *VERVE-101* in 10 patients with heterozygous FH, history of coronary revascularization, and a mean LDL-C of 193 mg/dL despite receiving maximally tolerated lipid lowering treatments. 10 patients (8 men and 2 women) received a single intravenous infusion of *VERVE-101* with the dosages of 0.1 (n = 3), 0.3 (n = 3), 0.45 (n = 3), and 0.6 mg/kg (n = 1). Nine patients were followed for 28 days and blood values of PCSK9 were decreased by 59% and 84% in those received 0.45 mg/kg dose and 45% in the patient received 0.6 mg/kg. LDL-C level was reduced by 39% and 48% in participants received 0.45 mg/kg dose and 55% in the participant received 0.6 mg/kg dose. A 55% reduction in LDL-C of the patient received 0.6 mg/kg persisted for six months. Transient rise in liver enzymes and transient infusion reactions were observed. During the follow-up, three cardiovascular events in two patients were observed. 5 weeks following dosing, one patient who received 0.3 mg/kg dose experienced a fatal cardiac arrest, which was not related to the drug since both patients had extensive atherosclerotic cardiovascular disease (ASCVD) and the independent safety bord claimed that these events probably related to underlying ASCVD diseases [67]. The other patient (received 0.45 mg/kg dose) experienced acute myocardial infarction one day after infusion and non-sustained ventricular tachycardia after a month after infusion. The independent safety claimed that these adverse events were not related to the drug and are consistent with their underlying cardiovascular diseases; therefore, they decided to continue the trial [60, 67].

### 4.3 ANGPTL3

In addition to *PCSK9*, targeting *ANGPTL3* can be considered as a valuable therapeutic approach for dyslipidemia management. A number of experimental research has revealed that ANGPTL3, which is secreted mainly in the liver, is able to inhibit lipoprotein lipase (LPL) and endothelial lipase (EL) activity, and its inactivation by mutations is associated with enhanced activity of LPL, causing lower levels of lipids and conveying protection against CVD [68, 69]. A monoclonal antibody drug, named evinacumab, with the ability to target ANGPTL3 has developed, and it has been demonstrated that this drug can reduce lipid levels through several mechanisms [68, 70], suggesting that targeting ANGPTL3 may be a compelling therapeutic option for the management of HoFH patients [71] and has an effect on LDL-C independent of *LDLR * [72].

Qiu *et al*. [73] utilized a lipid nanoparticle to deliver CRISPR-Cas9 mRNA to mice liver to knock down *ANGPTL3*. The efficacy of this method was appeared 38%, and the levels of ANGPTL3, LDL, and triglyceride (TG) were decreased 65%, 56%, and 29%, respectively, and these effects were lasted for 100 days. The authors found no off-target incidents in nine sites and moreover, no toxicity impact was found in the liver [73]. Lipid nanoparticles deliver gene editing through liver LDLR; therefore, it is not efficient to use standard lipid nanoparticles as a delivery system in patients with HoFH; nevertheless, Kasiewicz *et al*. [74] designed a new lipid nanoparticle system included N-acetylgalactosamine (GalNAc) with the ability to bind to the asialoglycoprotein receptor (ASGPR) to target ANGPTL3 in nonhuman primates with HoFH. The efficacy of this new technology was much higher than standard lipid nanoparticles [74].

Adenovirus vectors with the capacity to encode EB3 and guide RNA targeting *ANGPTL3* were generated by Chadwick *et al*. [75] so as to manage patients with dyslipdemia. After a week following the injection, 49%, 31%, and 19% reduction in levels of ANGPTL3 protein, blood TC, and cholesterol were detected, respectively. The investigators also found out that targeting *ANGPTLl3* could lower the blood levels of TG more than targeting PCSK9 or the combination of ANGPTL3 and PCSK9. At last, the authors evaluated the impact of targeting *ANGPTL3* in * LDLR*-negative mice, which resembles a proper model of HoFH and found a 56% and 51% reduction in TG and cholesterol, respectively, after two weeks after the injection. Interestingly, no reduction in bone marrow hematopoietic stem cells was appeared [75].

It is of importance to note that using drugs with the ability to target *ANGPTL3* may be accompanied by complications that should be paid attention to. Vupanorsen is an antisense oligonucleotide that target *ANGPTL3* in the liver. A randomized clinical trial reported an increase in liver function tests up to three times and also, hepatic fat fraction up to 76%. It is totally unclear that these complications are attributed to metabolic effects of the drug or off-target incidents due to *ANGPTL3* targeting [76]. Thus, adverse effects of drugs enabling to target *ANGPTL3* should be assessed more carefully. Of note, a very recent study conducted by Pennisi *et al*. [77], tried to evaluate whether intracellular *ANGPTL3* down-regulation can contribute to enhance in lipid contents within the hepatocyte. The authors inhibited *ANGPTL3* expression by silencing RNA in primary human hepatocytes and HepG2/LX-2 3-dimensional spheroids, and in Huh7, HepG2, and Hep3B2 cultured in 2 dimensions. Intracellular *ANGPTL3* down-regulation was associated with enhanced hepatic TG contents in all models. It was shown that *ANGPTL3* provoked lower values of intracellular deiodinase type 1 protein, leading to a decrease in beta-oxidation and as a result, enhance in TG content. These findings suggest that that the intra- and extracellular inhibition of ANGPTL3 will have different effects, a feature of importance when applying gene editing for *ANGPTL3* compared to antibodies against *ANGPTL3 * [77].

### 4.4 APOC3

APOC3 is known as a glycoprotein mainly synthesized in the liver. It is able to inhibit hydrolysis and catabolism of triglyceride-rich lipoproteins (TRLs), which contribute to higher values of plasma TG [78]. A cohort study revealed that those with homozygous loss of function of *APOC3* experienced a lower rise in post-prandial TG [79]. Guo *et al*. [80] developed a human-like model to assess the effects of *APOC3* inactivation on the lipid profile. The plasma levels of TG were reduced significantly in *APOC3* knockout hamsters on chow diet; nonetheless, high-density lipoprotein cholesterol (HDL-C) and total cholesterol values did not change significantly. Lipoprotein disc electrophoresis demonstrated an enhancement in LDL fractions and a decrease in very low-density lipoprotein (VLDL) fractions, pointing out that *APOC3* inhibition can increase the conversion of VLDL to LDL. The authors put hamsters on a high-cholesterol/high-fat diet to assess the association between *APOC3* and atherosclerosis and observed reduced levels of TG, total cholesterol, ApoB, and ApoE and enhanced levels of HDL-C and ApoA1. In summary, they concluded that APOC3 inhibition can exert cardioprotective effects through modifying lipid levels [80]. In another *in vivo* research, APOC3 protein expression was inhibited by CRISPR/Cas9 approach. While under a normal chow diet, only plasma levels of TG were decreased significantly, under a high-fat diet, plasma levels of TG, LDL-C, and total cholesterol (TC) were decreased significantly, highlighting that *APOC3* inactivation could impact cholesterol transport, and this impact is pronounced under a high-fat diet [81]. Collectively, *APOC3* can be considered as a suitable target for management of dyslipidemia.

### 4.5 Lipoprotein(a) [Lp(a)]

Accumulated evidence illustrated that lipoprotein (a) (Lp(a)) can act as an independent risk factor for CVDs [82]. Additionally, Lp(a) is carrier of oxidize phospholipids, contributing to the progression of atherosclerotic plaque [83]. Lp(a) is encoded by *LPA* gene, which produces in the liver and can bind to *ApoB100 * [84]. It has been reported that up to 90% variations in Lp(a) levels can be explained by variations in *LPA* gene [85]. Lp(a) concentration cannot be modified by lifestyle modifications or diet changes; hence, interventions should be implemented to change it [86]. An *in vivo* study sought to assess the capability of promoting *ApoB* mRNA editing for decreasing the risk of atherosclerosis. It was demonstrated that transfer of APOBEC1, which was encoded by recombinant adenovirus, enhanced *ApoB* mRNA editing in transgenic mice, leading to a remarkable decrease in plasma Lp(a) concentrations. The same findings were appeared when *ApoB* mRNA was edited in rabbits. *ApoB* gene editing in rabbits was correlated with decreased LDL concentrations [87]. A very recent study applied AAV-CRISPR to disrupt *LPA* transgene in the mice livers in order to decrease Lp(a) concentration. The outcome was satisfactory and Lp(a) levels decreased to a great extent and importantly, this approach was completely safe since no changes in liver function tests, overall body weight, cholesterol, and liver histology were observed [88].

## 5. Therapeutic Considerations 

We should optimize genome editing technologies efficacy and safety in order to bring them into clinical practices and assess their impacts on human. In this regard, choosing the best delivery tool is of great importance. Adenoviral vectors should not be considered to be used in clinical settings due to their safety problems and instead, AAVs are preferred [89, 90]; however, there are several limitations that should be tackled first. The main problem is that the size limitation of AAVs hinders the efficient delivery of base editors or SpCas9. The second problem is that using AAVs vectors will bring about extended expression of the delivered gene. Although this feature may seem beneficial, it can increase the incidence of off-target mutagenesis [75]. Using lipid nanoparticles as tools for the delivery can overcome the size and degradation limitations properly [91, 92].

The experimental studies that have investigated the applicability of genome editing for the management of dyslipidemia have reported low off-target mutagenesis incidents, but unfortunately, they are not fully reliable because next-generation DNA sequencing has some obstacles. First, its sensitivity is not high to detect rare mutations and these rare mutations may cause some problems. Second, DNA sequencing cannot assess the whole genome and only detects off-target incidents at particular sites. Several unbiased methods, in particular whole genome sequencing (WGS), have been proposed that are able to detect off-target mutagenesis precisely. WGS has been applied in several studies to find off-target incidents introduced by CRISPR/Cas9 in animals, plant, and human primary cells and the outcomes were promising. However, the main limitation of this method is that it is highly expensive [93].

## 6. Conclusions

Exploration of lipid-related genes contributed to the invention of new treatments with the ability to target these genes for the prevention of atherosclerosis; nonetheless, these medications should be administered frequently effective concentrations. Gene editing is potent to alter the genes expression for a long duration and exerts its long-term impacts on dyslipidemia. Noteworthy, due to permanent changes in genome, it is of great importance to evaluate its safety in the long run.

## Abbreviations

CVD, cardiovascular disease; PCSK9, proprotein convertase subtilisin/kexin type 9; TALEN, transcription activator-like effector nuclease; ZFNs, zinc-finger nucleases; CRISPR-Cas9, clustered regularly interspaced short palindromic repeats-CRISPR-associated protein 9; nCas9, nickase Cas9; dCas9, dead Cas9; PAM, protospacer-adjacent motif; C, cytosine; T, thymine; G, guanine; A, adenine; pegRNA, primary editing guide RNA; HDR, homology-directed repair; KRAB, Krüppel-associated box; LDLR, LDL receptor; ANGPTL3, Angiopoietin-like protein 3; APOC3, apolipoprotein C3; FH, familial hypercholesteremia; ApoB, apolipoprotein B; CHD, coronary heart disease; HoFH, homozygous familial hypercholesteremia; Cas9n, Cas9 nickase; sgRNA, paired single-guide RNAs; AAV, adeno-associated virus; SaCas9, Staphylococcus aureus Cas9; TBG, thyroid hormone-binding globulin; AST, aspartate aminotransferase; ALT, alanine aminotransferase; LPL, lipoprotein lipase; EL, endothelial lipase; GalNAc, N-acetylgalactosamine; ASGPR, asialoglycoprotein receptor; TRLs, triglyceride-rich lipoproteins; Lp(a), lipoprotein (a); WGS, whole genome sequencing.
